# A Retrospective Pilot Study for Preoperative Screening to Prevent Iatrogenic Cervical Spinal Cord Injury

**DOI:** 10.7759/cureus.12550

**Published:** 2021-01-07

**Authors:** Anthony Diaz, Christopher Chin, Stephen S Burks, David McCarthy, Christina Matadial, Howard B Levene

**Affiliations:** 1 Department of Neurological Surgery, University of Miami Miller School of Medicine, Miami, USA; 2 Department of Neurological Surgery, University of Pittsburgh Medical Center, Pittsburgh, USA; 3 Department of Anesthesiology, University of Miami Miller School of Medicine/Miami Veteran Affairs Healthcare System, Miami, USA; 4 Research, Miami Veterans Hospital, Miami, USA; 5 Neurological Surgery, Levene Neurosurgical Consulting, Inc, Miami, USA

**Keywords:** preoperative clearance, cervical hyperextension, central cord syndrome, cervical spine injury, iatrogenic

## Abstract

Purpose: The preoperative medical clearance process is well established to screen for medical comorbidities and therefore must be thorough. However, screening for potential cervical spine disease is often overlooked. In older surgical candidates, the presence of cervical spondylosis can increase risk of iatrogenic cervical spine injury during prolonged neck extension in non-spinal surgeries. We present a standard protocol for cervical spine clearance and a novel sustained neck extension maneuver through a retrospective case series.

Methods: Sixty-three consecutive cases that underwent preoperative cervical clearance between April 2012 and December 2019 were reviewed. Referral for clearance occurred through the department of anesthesiology after concerning radiographic or physical exam findings were noted. A standard preoperative screening protocol with a sustained one-minute neck extension maneuver was implemented. Recommendations were made for standard neck precautions with or without neuromonitoring or for cervical spine decompression surgery prior to the planned procedure.

Results: There were 25 patients with symptoms of myelopathy, 11 with radiculopathy and 13 with neck pain at baseline. Cervical spondylosis was observed in 51 patients, cervical canal stenosis in 29 and cervical myelomalacia in six. Fifty-seven patients underwent neck extension exam and 25 exhibited new or worsening symptoms. Myelopathic symptoms and radicular pain at baseline and positive Hoffman’s and Spurling’s sign, independently, were significantly associated with a positive neck extension exam (p<0.05). Fourteen patients were recommended for cervical decompression prior to planned procedure.

Conclusions: Our preoperative cervical spine clearance protocol is safe and may aid in identifying patients susceptible to iatrogenic cervical spine injury.

## Introduction

Preoperative medical clearance is accepted as essential for minimizing surgical risk. Traditionally, preoperative medical evaluation identifies and stratifies risk factors associated with perioperative complications. Often performed by an anesthesiologist or primary care physician, preoperative screening includes a critical review of the patient’s age, exercise capacity, medications, weight, alcohol and smoking use, pulmonary and cardiac risk, and additional factors depending on the indicated procedure [1-5]. Laboratory and radiologic studies play a crucial role and include, but are not limited to, blood work, electrocardiogram, chest radiograph, and pulmonary function tests [6-8].

While the preoperative medical clearance process can be thorough, screening for potential cervical spine disease is often overlooked. In older surgical candidates, cervical spondylosis with subtle or overt myelopathy is relatively common [9]. During a prolonged surgical procedure, patients may be exposed to mechanical and gravitational forces from neck positioning (eg, cervical hyperextension or hyperflexion) that when coupled with an existing cervical spine disease, may increase the likelihood of an iatrogenic cervical spinal cord injury [10]. In addition, cervical spine disease and cervical spine instability have been strongly associated with difficulty and complications in airway evaluation and management, potentially exacerbating the risk for injury [11].

There are multiple case reports in the literature that have reported iatrogenic cervical cord injuries for non-spinal surgeries in patients with previously undiscovered cervical spondylosis [12,13]. Interestingly, in most reported cases, patients experienced unremarkable preoperative neck and neurologic evaluations that failed to elicit myelopathic symptoms [12]. It is possible, then, that current neck assessment maneuvers might not be sensitive enough to screen patients who are at risk of developing an iatrogenic cervical cord injury during periods of prolonged neck extension. Additionally, despite devastating reports of these injuries and literature urging for preoperative cervical spine clearance, there remains no standard protocol.

We present a retrospective case series of patients for which preoperative cervical clearance was requested from anesthesia. A standardized protocol was implemented to ensure operative safety. In addition, we propose a sustained one-minute neck extension exam that mimics prolonged deformity, compressing the cord and vessels long enough to elucidate subtle and overt myelopathic symptoms. This maneuver mimics neck positioning during intubation and a variety of different surgical procedures requiring prolonged neck extension (ENT, dental, etc.) for a better assessment of the risk of an iatrogenic cervical cord injury.

## Materials and methods

Patient selection

Following local institutional board approval (IRB#5504.03), we retrospectively reviewed patients for which a single neurosurgeon was consulted for pre-operative cervical clearance. We identified patients from April 2012 to December 2019 at a Veteran Affairs Hospital. We included all patients for whom neurosurgery was consulted for pre-operative cervical spine clearance and were subsequently examined by a single neurosurgeon. There were 63 cases that met this criterion.

Screening process

**Figure 1:**
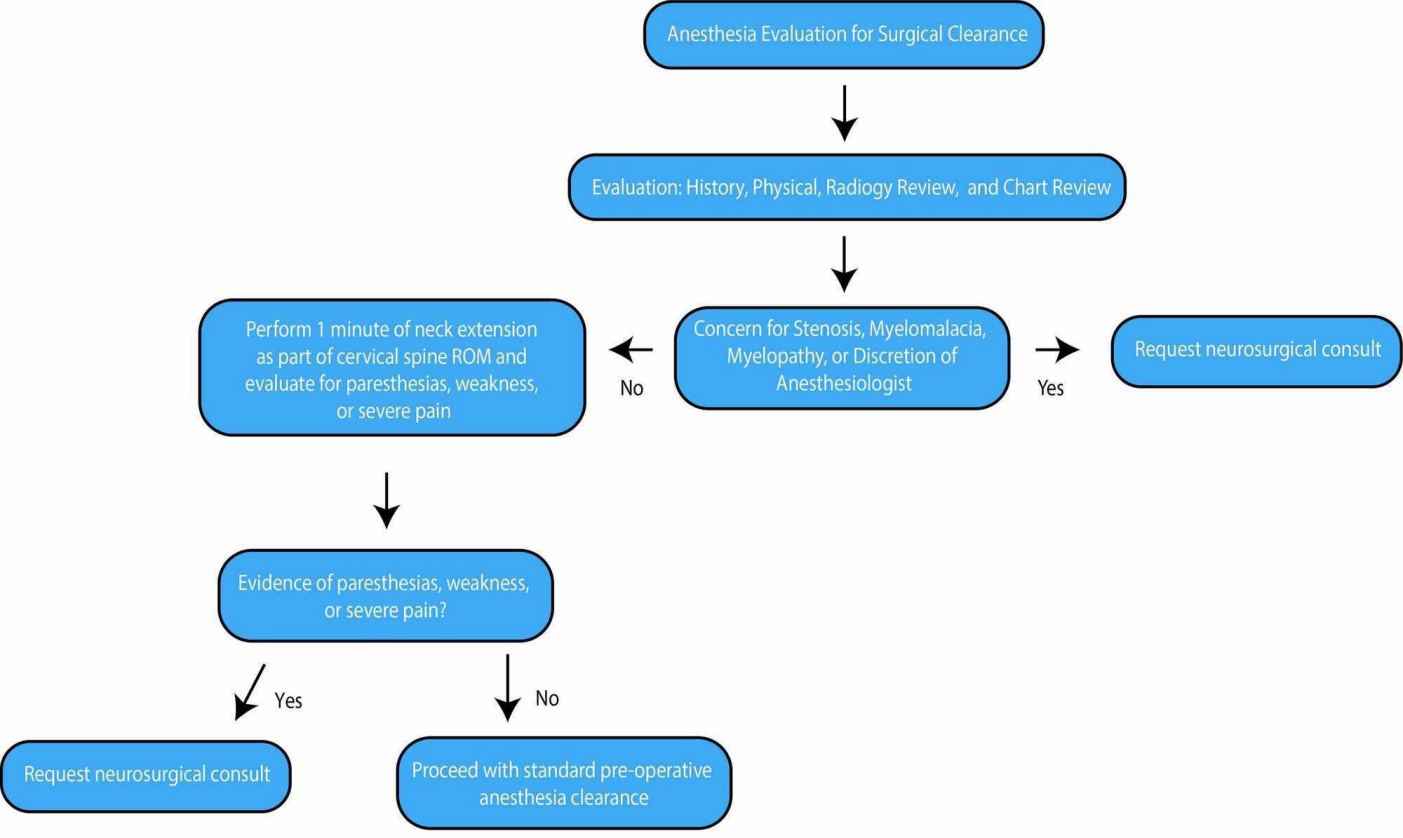
Cervical Clearance Protocol Flow chart illustrating decision making at each stage of the preoperative cervical clearance evaluation.

Patients were typically referred for preoperative cervical spine clearance through the department of anesthesiology at our institution. If cervical pathology was present on radiographic imaging and the surgery required a degree of neck extension/manipulation the patient was referred to Neurosurgery. After the neurosurgical team was consulted for cervical clearance, a standard protocol was implemented (Figure 1). A member of the neurosurgical team obtained relevant history and physical exam. Available imaging was examined for evidence of cervical spondylosis, myelomalacia and other radiographic findings. Standardized preoperative cervical screening consisted of evaluating for signs of myelopathy, radiculopathy, and neck pain in the reported history, as well as obtaining a detailed physical exam including deep tendon reflexes, motor strength, sensation, and Spurling’s and Hoffman’s test. In addition to cervical range of motion, a sustained neck extension maneuver was employed during the physical exam evaluation. Specifically, patients were asked to sustain full extension of the neck for one minute and were then assessed for evidence of new or worsening myelopathic or radicular symptoms, indicating a positive test. In patients with clear signs of severe myelopathy or radiculopathy on physical exam or in patients unable to perform neck extension, the sustained neck extension exam was deferred/not performed.

Post-screening recommendations

Post-screening recommendations were made with consideration of the patient’s history, baseline symptoms, physical exam findings, severity of imaging findings, extent and necessity for neck manipulation during planned procedure and urgency of the planned surgery as illustrated in Figure 1. In patients with a benign clinical exam and non-concerning imaging findings, recommendations were made for standard neck precautions, such as minimizing neck extension and consideration of fiberoptic intubation (FOI). In patients with mildly concerning physical exam findings, able to tolerate neck extension during sustained neck extension exam, and with mildly degenerated or stenotic spines seen on imaging were recommended for standard neck precautions with the addition of somatosensory evoked potential (SSEP) monitoring. However, in patients with more severe baseline symptoms and with new or worsening myelopathic or radicular symptoms during sustained neck extension exam or in patients unable to tolerate neck extension exam due to severity of symptoms, recommendations were made for decompressive cervical spine surgery before the originally planned procedure. In certain cases where the planned procedure was critical and/or urgent and cervical spine decompression was not possible, recommendations for neuromonitoring and standard neck precautions were made while alerting the patient to the cervical spine-related risks of surgery.

Statistical analysis

Data was analyzed using Statistical Package for Social Sciences (SPSS) version 25.0 (IBM Corp., Armonk, NY, USA) . All values are reported as mean ± standard deviation unless stated otherwise. Cases were grouped based on whether they had a positive or negative neck extension exam. Two-tailed student t-tests were performed to evaluate for associations between radiographic findings, clinical exam findings and age and chi-square analyses were used to evaluate categorial variables among both extension exam outcome groups. A p-value < 0.05 was considered significant.

## Results

Among the 62 patients that were referred for cervical spine clearance, one patient was screened on two separate instances after request from two separate services, and therefore each visit was treated as an individual case. As shown in the demographics data in Table 1, 95% (n=60) of our cohort were male and the mean age at time of evaluation was 62.6 ± 10.2 years. The gender disproportionality reflects the patient population of our institution, a VA hospital. A list of all services from which pre-operative cervical clearance was requested is presented in Table 2, with otolaryngology (43.3%), orthopedics (19.0%) and cardiothoracic surgery (11.1%) being the three most predominant. Shoulder surgeries (n=8), oral tumor/cancer resections (n=6) and coronary artery bypass grafts (CABG) (n=6) were the most common procedures for which spine clearance was requested. 

**Table 1:** Demographics Data

Demographics data	
	Number
Number of patients	62
Number of consults	63
Age*	62.6 ± 10.2 (35-88)
Sex (F/M)	3/60
*Mean ± SD (range)	

**Table 2:** Services Requesting Preoperative Evaluation Abbreviations: SCC (Squamous Cell Carcinoma), THA (total hip arthroplasty), TSR (total shoulder replacement), CABG (coronary artery bypass graft) *Values are number (percent)

Services requesting preoperative evaluation		
Consulting Service	N=63^*^	Procedures
Otolaryngology (ENT)	27 (43.3)	Oral tumor/SCC resections, laryngoscopy/laryngectomy, vocal cord biopsy, tracheostomy, thyroidectomy, palate surgery, septoplasty, hearing aid surgery, mastoid surgery
Orthopedics	12 (19.0)	THA, TSR, rotator cuff tear, knee surgery
Cardiothoracic Surgery	7 (11.1)	CABG, aortic valve replacement
Urology	5 (7.9)	Penile implant, urosphincter surgery, cystoscopy
General Surgery	4 (6.3)	Whipple, cholecystectomy, robot prostatectomy, abdominal mesh
Gastroenterology	3 (4.8)	Endoscopy
Ophthalmology	2 (3.2)	Cataract removal
Vascular Surgery	2 (3.2)	Carotid endarterectomy
Plastic Surgery	1 (1.6)	Skin flap removal surgery

Findings on evaluation

Baseline neurologic symptoms and physical exam findings are listed in Table 3. Forty-three cases had symptoms at baseline, such as myelopathy (n=25), radiculopathy (n=11), neck pain (n=13), paresthesias (n=12), or other (n=20), such as muscle weakness, extremity pain, and gait disturbances. Five cases exhibited hyperreflexia, seven cases had positive Spurling’s sign and seven separate cases had positive Hoffman’s sign. Twenty cases had no neurological complaints/symptoms at baseline or positive physical exam findings but had evidence of cervical spine pathology on imaging. 

**Table 3:** Symptoms At Baseline and Physical Exam Findings

Symptoms at baseline and physical exam findings	
	Number
Patients without baseline symptoms	20
Symptoms at baseline	43
-Myelopathy	25
-Radiculopathy	11
-Neck Pain	13
-Other^*^	20
Hyperreflexia	5
Positive Hoffman's sign	7
Positive Spurling’s sign	7
*Includes motor weakness, extremity pain and gait disturbances	

We observed 51 (81.0%) cases with evidence of cervical spondylosis, 29 (46.0%) with cervical canal stenosis, and six (9.5%) with cord signal intensity changes suggestive of myelomalacia in Table 4. There were 15 cases with cervical spondylosis on imaging who did not have any baseline symptoms. One patient with no radiographic findings had complaints of intermittent mild radiculopathy. The mean age of patients with vs without radiographic evidence of cervical spondylosis was 62.8 ± 10.7 years vs 60.6 ± 7.5 years (p-value = 0.056). 

**Table 4:** Sustained Neck Extension Findings

Sustained neck extension findings	
	Number
Underwent neck extension*	53 (88.3)
Did not undergo neck extension*	7 (11.6)
Symptoms with neck extension (N=22)	
-Paresthesias	11
-Sensory deficits	10
-Radicular pain	7
-Neck/Shoulder pain	3
*Values are number (percent)	

Neck extension findings

Upon initial evaluation of baseline symptoms, radiographic findings, and physical exam, and with consideration of planned operative procedure, 57 (90.5%) of the 63 cases were designated for neck extension exam. Six cases did not undergo sustained neck extension exam due to clear signs of severe myelopathy on physical exam, such as clonus, brisk hyperreflexia, or severe motor weakness or due to inability to extend neck. One case had sustained neck extension exam bypassed due to having very minimal baseline symptoms and radiographic findings as well as undergoing a procedure that did not require significant neck extension or manipulation.

With neck extension sustained for one minute, we elicited new onset or worsening symptoms in 25 (43.9%) of the 57 cases that had the exam. Twenty-two cases experienced paresthesias with neck extension, 12 of which were new-onset. Two patients, without any neurologic complaints at baseline or physical exam findings, endorsed new-onset paresthesias in the upper extremities that resolved with neutral position. Radiculopathy was elicited in seven cases, two of which only had minor neck pain at baseline. No statistical correlations were observed between age and presence of myelopathic or radicular symptoms either at baseline or with neck extension.

To explore for predictors of outcome, specifically with regards to sustained neck extension exam outcomes, we grouped the 57 cases that underwent the sustained neck extension exam based on whether they had positive findings or not in Table 5. Statistical associations between reported baseline symptoms/physical exam findings and outcome groups were analyzed using chi-square analysis. We observed that the presence of baseline myelopathic symptoms and radiculopathy as well as a positive Hoffman’s and Spurling’s sign, independently, were significantly associated with the presence of neck extension findings through chi-square test of independence, χ2(2, N=57)=12.1, p=0.0005, χ2(2, N=57)=6.4, p=0.012, χ2(2, N=57)=4.2, p=0.039, and χ2(2, N=57)=5.6, p=0.017, respectively. 

**Table 5:** Baseline Findings Grouped Based On Presence or Absence of Neck Extension Findings

Baseline findings grouped based on presence or absence of neck extension findings			
Baseline Symptoms/ Physical Exam Results	Cases with neck extension findings (N=22)^*^	Cases without neck extension findings (N=31)^*^	P-value
Neck/shoulder pain	5 (22.7)	7 (22.6)	0.99
Paresthesia	10 (45.5)	1 (3.2)	0.00019
Radicular pain	8 (36.4)	2 (6.5)	0.0061
Sensory deficits	5 (22.7)	2 (6.5)	0.085
Motor deficits	1 (4.5)	5 (16.1)	0.19
Hyperreflexia	3 (13.6)	1 (3.2)	0.16
Positive Spurling's	6 (27.3)	1 (3.2)	0.011
Positive Hoffman's	4 (18.2)	1 (3.2)	0.066
*Values are number (percent)			

Post-screening recommendations and outcomes

Table 6 lists recommendations made after cervical clearance evaluation. A total of 15 patients had significant changes to their initial surgical plan. Fourteen cases were recommended for cervical decompression and one case had recommendations for transition from aortic valve replacement to a temporary percutaneous balloon valvuloplasty for subsequent laminectomy and fusion. Ten cases that were recommended for cervical decompression had a positive sustained neck extension exam and three did not undergo neck extension exam secondary to severe myelopathic or radicular symptoms coupled with severe radiographic findings. One case with an initial negative cervical extension exam was recommended for decompression after a fall precipitated worsening baseline neurologic symptoms immediately prior to the planned operative procedure.

**Table 6:** Post-Screening Recommendations

Post-screening recommendations			
Post-Screening Recommendations	Cases with neck extension findings (N=22)	Cases without neck extension findings (N=31)	Cases without neck extension exam (N=7)
Standard neck precautions alone	0	19	1
Standard neck precautions with neuromonitoring	22	12	6
Decompression	7	1	4
Alternate approach^*^	1	0	0
*Recommendation for percutaneous approach for aortic valve replacement			

All 25 patients with a positive neck extension exam had recommendations for standard neck precautions with neuromonitoring, regardless of the planned procedure. Standard neck precautions alone, such as minimizing or avoiding neck extension, were recommended in 21 cases, all of which had very benign clinical exams and non-concerning imaging findings. In 12 cases with a negative neck extension exam but with concerning imaging findings and/or requiring significant neck manipulation, the addition of intraoperative SSEP monitoring was recommended. In general, patients with mild to moderate concerning physical exam findings and/or with evidence of mildly degenerated or stenotic spines in imaging were recommended for neuromonitoring.

Among those recommended for cervical decompression, seven patients underwent decompression without negative sequelae and with significant improvement of myelopathic and radicular symptoms. Two patients are currently undergoing further evaluations and five were lost to follow-up after cervical spine evaluation. All with patients with recommendations for either standard neck precautions alone or with the addition of neuromonitoring recovered from their respective procedures without any neurological sequelae.

Cases

**Figure 2:**
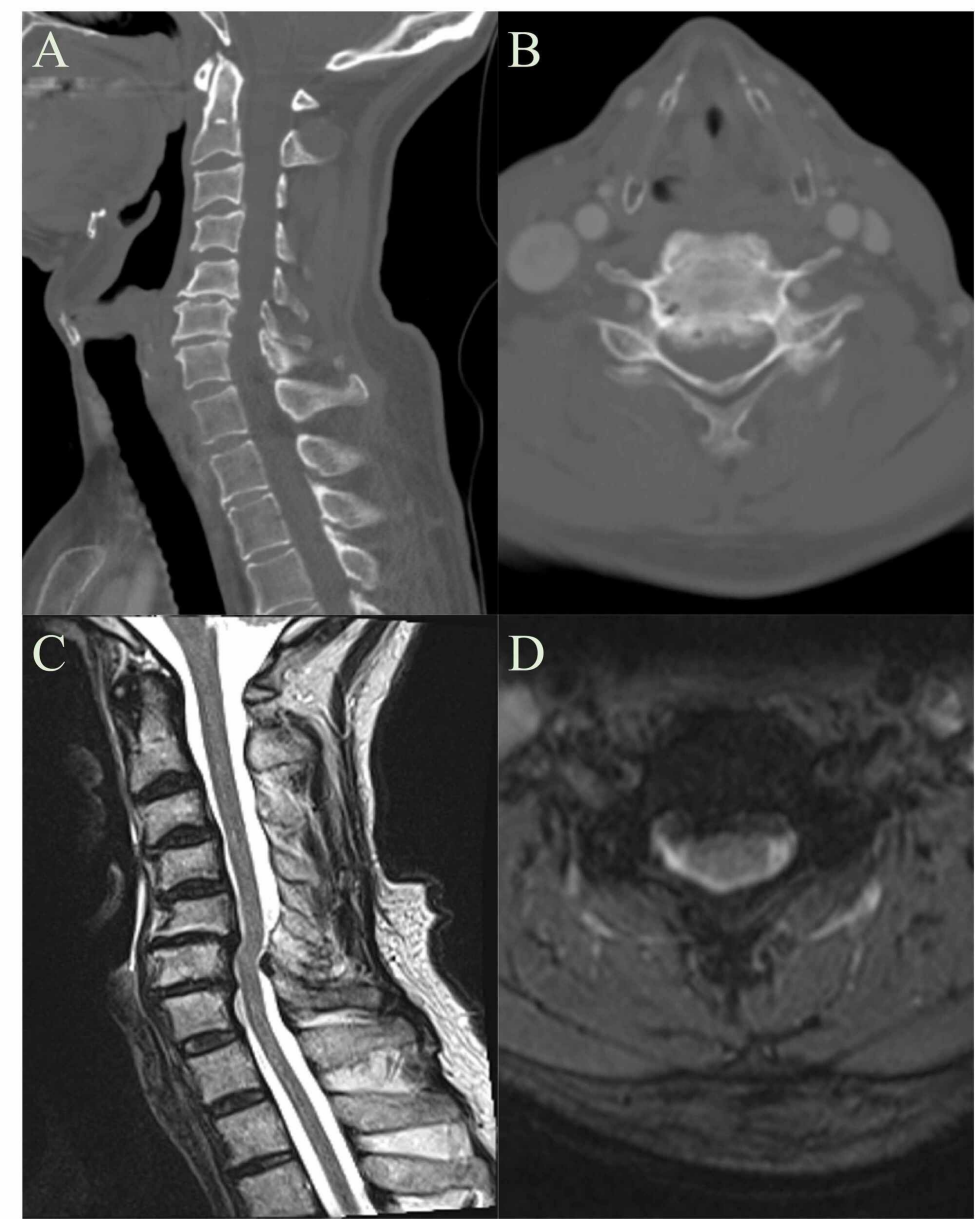
Patient 1 CT and MRI of the Neck A: Patient 1 CT Neck Sagittal View B: Patient 1 CT Neck Axial View C: Patient 1 MRI Neck Sagittal View D: Patient 1 MRI Neck Axial View Evidence of cervical stenosis is seen at C6-C7 (Cervical Vertebrae 6-7).

Patient 1 was a 58-year-old male that was scheduled for mandibulectomy, free flap, and complex vestibuloplasty/oral cavity reconstruction for left anterior floor of mouth squamous cell carcinoma with an Ear, Nose, and Throat (ENT) specialist. The patient had a history of chronic neck pain that developed from a diving injury. Computed Tomography (CT) and Magnetic Resonance Imaging (MRI) of the neck showed evidence of cervical stenosis at C6/7 (Figure 2). No significant findings were observed during physical exam and sustained neck extension maneuver did not elicit new or worsening symptoms. The patient was cleared for ENT procedure with recommendations for standard neck precautions. The patient recovered from the procedure without any new neurological symptoms or complications.

**Figure 3:**
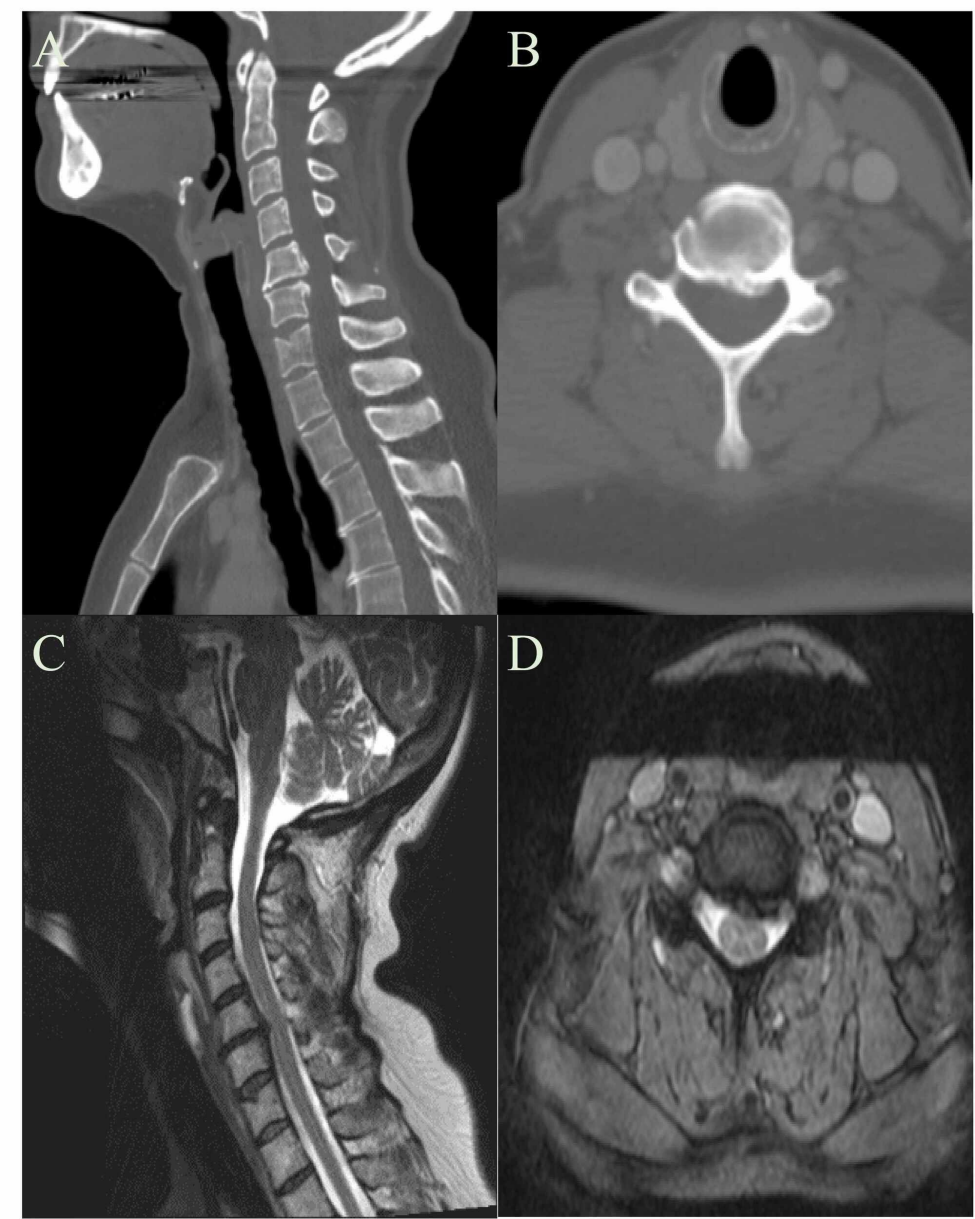
Patient 2 CT and MRI of the Neck A: Patient 2 CT Neck Sagittal View B: Patient 2 CT Neck Axial View C: Patient 2 MRI Neck Sagittal View D: Patient 2 MRI Neck Axial View A disc osteophyte complex with narrowing of the spinal canal is seen at C5-6 (Cervical Vertebrae 5-6).

Patient 2 was a 51-year-old female that was scheduled for squamous cell carcinoma resection with ENT. Planned surgery included vestibuloplasty, oral cavity reconstruction, tongue resection, and free flap. CT and subsequent MRI of the neck showed a prominent disk osteophyte complex at C5/6 (Figure 3) with no prior history of surgery or prior complaints of neck or arm pain. Neurosurgery was consulted, and physical exam revealed hyperreflexia (3+ throughout) and Hoffman’s sign bilaterally. Due to the severity of the patient’s baseline symptoms and physical exam, sustained neck extension maneuver was bypassed and the patient was recommended to undergo anterior cervical discectomy and fusion (ACDF) immediately prior to cancer resection under a single intubation. The patient demonstrated excellent recovery from both procedures without any neurologic deficit.

**Figure 4:**
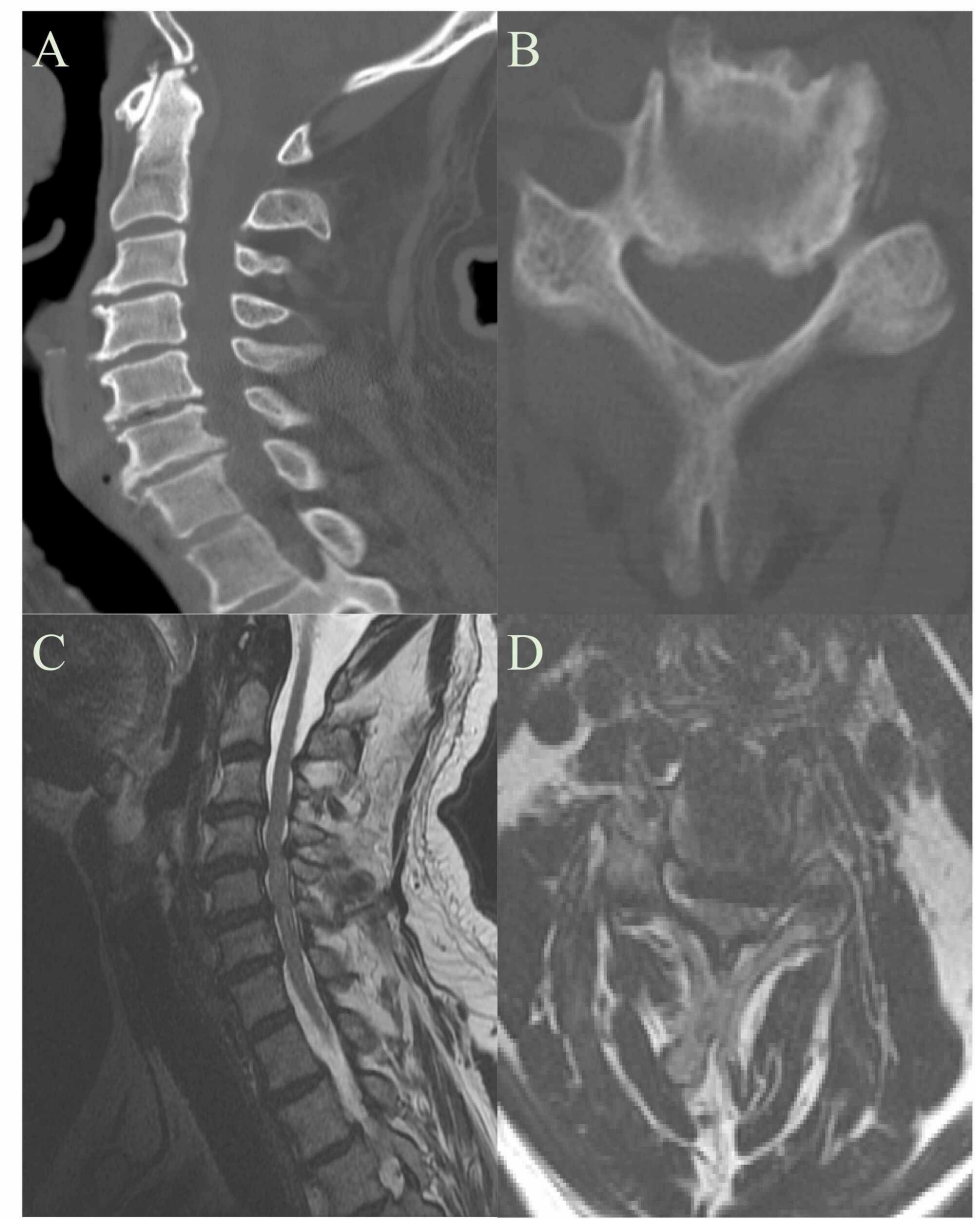
Patient 3 CT and MRI of the Neck A: Patient 3 CT Neck Sagittal View B: Patient 3 CT Neck Axial View C: Patient 3 MRI Neck Sagittal View D: Patient 3 MRI Neck Axial View Multilevel cervical stenosis can be seen in A and C.

Patient 3 was a 59-year-old male scheduled for spine surgery at an outside hospital due to symptomatic multi-level cervical stenosis, as shown in T2 MRIs in Figure 4. Symptoms included myelopathic gait, hand numbness and clumsiness, and upper extremity weakness. When undergoing cardiac clearance, a positive stress test lead to a cardiac catheterization that revealed severe coronary artery disease. Neurosurgery was consulted to decide if a CABG was safe in the setting of patient’s spine disease. Despite worsening paresthesias to right arm with sustained neck extension maneuver and positive Hoffman’s sign, the necessity for CABG outweighed the need for cervical decompression. Patient underwent CABG x 2 with recommendations for standard neck precautions and intraoperative SSEP monitoring and recovered remarkably well without neurological sequelae.

## Discussion

It is well accepted that medical clearance be performed preoperatively to reduce surgical risk. Cervical spine clearance prior to surgery should also be an important clearance tool to reduce risks, especially in vulnerable patients. We propose adding the maneuver of holding the neck in extension for one minute in addition to range of motion during cervical examination in patients being assessed for risk of cervical spine injury prior to undergoing procedures that require significant neck manipulation. This test conceptually reproduces compression in the same way a Phalen's test compresses the median nerve in carpal tunnel assessment. The exam can be easily performed with minimal effort during a standard pre-operative evaluation.

Case for cervical spine clearance

Underlying cervical spondylosis, cervical spine tumors, and atlanto-axial subluxations may predispose a patient to positional injury. Iatrogenic cervical spinal cord injuries can result in central cord syndrome (CCS), Brown-Sequard syndrome, quadriparesis, quadriplegia, and even death. These injuries can occur when underlying anatomical spine pathologies, such as herniated discs, osteophytes, and thickened ligaments among others, impose additional compressive forces on the cervical cord and spinal vessels during prolonged hyperextension [14]. In a closed claims analysis that reviewed 48 claims of cervical cord, root, and/or bony spine injuries recorded in the American Society of Anesthesiologists Closed Claims database, Hindman et al. [15] reported 13 cases associated with noncervical spine surgeries. Among these cases, cervical spondylosis was present in nearly all cases (85%) and neck positioning was recorded as the most probable contributor to injury in nearly half (46%) of the cases. 

Iatrogenic CCS, quadriplegic injuries, and various other neurologic sequelae have been reported following a wide variety of non-spinal procedures, which include dental extraction [16], thyroidectomy [12], parathyroidectomy [17], adenotonsillectomy [18], CABG [19-22], and knee arthroplasty [23]. In a case report by Xiong et al. that presented a patient who developed quadriplegia after a subtotal thyroidectomy, a summary of 14 previously published cases with iatrogenic quadriplegia following non-spinal surgeries was outlined [12]. Among the 14 cases, 64% had evidence of cervical spondylosis, 67% were seniors (60 years or greater), 92% were male, 77% had permanent disability and 2 patients died during short-term follow-up. Preoperatively, none of the patients complained of radicular pain, neck pain, or any neurologic symptoms and all had a normal range of motion of the neck and neurologic exam. In nearly all the cases evaluated in Hindman et al. [15], cervical cord injuries occurred in patients with stable cervical spines, absent preoperative symptoms, and with severe underlying cervical spondylosis. These highlight the importance of careful preoperative screening for cervical spondylosis, especially in the older patient population. In addition, they also highlight the limitations with current neck exam maneuvers for screening asymptomatic patients at risk of developing an iatrogenic cervical cord injury.

Data on the sensitivity and specificity of standard physical exam maneuvers for detecting degenerative cervical myelopathy has not been fully elucidated. There are a few recent studies that have looked at the validity of upper extremity reflexes and Hoffman sign. In a systematic review of 201 patients presenting to spine specialists from three separate cross-sectional studies, Fogarty et al. [24] reported the positive likelihood ratio (ie, likelihood that a patient with cervical myelopathy will test positive) of a positive Hoffman’s sign for cervical myelopathy to range between 1.8 - 6.0 with a combined value of 2.6 (95% CI 1.8-3.9) and a negative predictive value (ie, probability that a patient with a negative test does not have cervical myelopathy) of 70%. This represents only a modest contribution to the overall diagnostic picture of a patient with degenerative cervical myelopathy. They concluded that a positive Hoffman is unlikely to be clinically useful without the other components of a thorough evaluation (eg, complete history and other physical exam findings). In addition, all patients included in these studies presented to a spine specialist with neck pain, potentially increasing the probability of a positive finding. Grijalva et al. [25] found a similar negative predictive value of 72.5% with a positive likelihood ratio of 1.21 (95% CI 0.91-1.61) for cervical cord compression. In both studies, about 40% of patients with confirmed cervical pathology had a negative exam, highlighting the low sensitivity for cervical myelopathy.

There is consensus in the anesthesia literature that most iatrogenic cervical cord injuries in non-spinal procedures result from prolonged deformation and/or impaired perfusion of the cord as opposed to the brief periods of small deformations that occur during intubation. The sustained neck extension maneuver that is proposed in this article allows for compression of the cord and vessels long enough to elucidate subtle and overt myelopathic symptoms in patients that have underlying cervical spondylosis, while conscious and under direct supervision. Therefore, we believe that a sustained neck extension maneuver will better mimic a prolonged deformity during an operative procedure as compared to current provocative signs, such as range of motion, Hoffman’s sign, and other reflex exams. Mechanically, this is similar to the Phalen’s test, where the median nerve positioned to be compressed for up to a minute to elucidate a neurological finding.

Cervical spondylosis should be considered in older patients undergoing any surgical procedure as it can affect up to 90% of men older than 50 years and women older than 60 years [26]. In addition, the cases described above highlight the importance of careful preoperative screening, even amongst patients who do not voluntarily express concern for myelopathic or radicular symptoms prior to surgery. We observed that among those with radiographic signs of cervical spondylosis, 29% reported very minimal to no baseline symptoms. Interestingly, we also observed that in 14 separate instances, patients developed new-onset myelopathic symptoms after sustained neck extension maneuver. Two of these patients had no complaints of baseline symptoms and subsequently were found to have evidence of cervical spondylosis on imaging. These findings provide further evidence that degenerative cervical changes can be present in an asymptomatic elderly patient, increasing their risk of an iatrogenic cervical cord injury with prolonged neck extension. Therefore some argue that neurophysiologic and clinical monitoring of patient positioning using SSEP monitoring should be integrated in any surgical procedure in severely elderly patients to detect and prevent position-related neuropathy [27,28].

Our cohort

Using our standardized preoperative cervical clearance protocol, we evaluated 51 cases with cervical spondylosis, 29 cases with cervical canal stenosis, and six cases with cervical myelomalacia for cervical spine clearance. In most instances, cervical decompression was not necessary and recommendations for careful neck manipulation alone or with neuromonitoring were made. Patients that were evaluated preoperatively by our service and subsequently underwent respective surgical procedures with our recommendations, faired significantly well without immediate evidence of neurodegenerative changes. Similarly, patients who elected for cervical decompression per our recommendations had significant improvement in baseline neuropathic symptoms.

Significant changes to the original planned operative course were mostly observed among patients with positive sustained neck extension findings. We also observed that a greater percentage of patients with baseline myelopathic symptoms (73%) and radiculopathy (80%), among all other symptoms, had positive sustained neck extension exams. In fact, the presence of myelopathic symptoms and radiculopathy at baseline as well as a positive Hoffman’ and Spurling’s sign on physical exam were significantly associated with a positive neck extension exam. Therefore, the presence of these clinical findings during preoperative evaluation can aid in increasing the pre-test probability of a patient having a positive sustained neck extension exam and potentially requiring significant alterations to the originally planned operative course. The sustained neck extension maneuver is a simple and safe provocative maneuver that can be easily adapted into the anesthesiologist’s toolbox in preoperative clearance evaluations without the need for complex maneuvers. This type of test will alert the anesthesiologist to the potential risk of neck extension for certain patients and may guide the anesthesiologist to look towards intubation techniques that limit neck extension (eg, McGrath or Glidescope).

Finally, patients with spinal cord injury often require extensive medical attention and resources due to the propensity of these injuries for lengthier hospital stays, long-term disability, and high frequency of hospital readmission. While the literature is limited in regard to cost analysis of spinal cord injury (SCI) care, it is clear that long-term management of SCI is associated with a significant financial burden in addition to the long-term disabilities and comorbidities incurred [29,30]. Therefore, adapting a cervical spine clearance protocol for preoperative evaluation of patients with increased risk of iatrogenic SCI could aid in reducing postoperative spinal cord complications and associated healthcare costs.

Limitations

A significant limitation of our study is that we are attempting to avoid an already rare complication, but potentially devastating. Due to the relatively small sample size, we have no way to assess if we in fact avoided an iatrogenic complication. It is possible that any percentage (0-100%) of the 15 patients for whom we made recommendations for either spine surgery prior to the original planned procedure or for whom their planned procedure was altered avoided a post-operative neurological complication. Similarly, patients undergoing SSEP monitoring may or may not have avoided a complication. Our sample size may have been too small to have identified a patient for whom intraoperative changes (eg, raising the blood pressure, repositioning the neck) were required due to SSEP monitoring during surgery. In the literature, there are only a few cases, specifically mentioned, which suggests the incidence of this injury may be low and our patient cohort was not powered enough to detect the avoidance of a complication. However, this does not obviate the need to be proactive in avoiding known complications and nothing we have proposed is outside the bounds of good conservative spine and anesthesia practice. Lastly, future studies are needed to evaluate the sensitivity of the sustained neck extension exam for cervical myelopathy. This can be performed through a prospective study of all patients evaluated by anesthesia for preoperative clearance. 

## Conclusions

We present a new protocol for preoperative cervical clearance along with our experience through a retrospective case series. No similar study has been published in the literature. In addition, we present a simple, novel sustained neck extension maneuver that reproduces compression of the spinal cord and mimics neck position during intubation and surgery. We observed that among patients who underwent the sustained cervical neck extension maneuver, baseline symptoms of paresthesias and radicular pain as well as positive Hoffman’s and Spurling’s sign were significantly associated with the presence of a positive neck extension exam. Additionally, all patients who had a significant change in initially planned operative course had either positive neck extension findings or were unable to undergo neck extension exam secondary to severe myelopathic or radicular symptoms. All patients who were screened and proceeded with our recommendations recovered without neurological complications. We hope that clinicians will find these recommendations valuable in the prevention of iatrogenic cervical spine injury related to non-spinal surgeries.
